# Bibliographical Notices

**Published:** 1856-10

**Authors:** 


### BIBLIOGRAPHICAL NOTICES.

*Human Physiology, Statical and Dynamical; or, The Conditions and Course of the Life of Man.* By John William Draper, M.D., LL. D., Professor of Chemistry and Physiology in the University of New York. *Illustrated with nearly* 300 *wood engravings.* New York: Harper & Brothers. 1856.

The position held by Professor Draper, as a distinguished original investigator, as well as author and teacher, has led us to expect much from this volume; and, in the anticipation that it would possess decided merits, we have not been disappointed.

It is "the text of the Lectures on Physiology which the author has given for many years in the University," to "classes of many hundreds." No haste has, therefore, enfeebled its preparation; but it contains the deliberate views of a teacher, experienced both in the matter of his study and in its expression. We may view it, fairly, in a three-fold aspect; first, as a Text book; secondly, as a Compend of the Facts of the Science discussed; and lastly, as a Philosophical or Methodological contribution to Science, in a wider sense.

As a text book, we should think it remarkably well adapted for success. Its general style is, in comparison with the others accessible to students in this country, especially agreeable and interesting; more so, perhaps, than that of any other now in use. Valentin's work, to which the treatise of Professor Draper bears, in style and manner, the most resemblance, is too cumbrous; and the same may be now said of Carpenter's, notwithstanding their unsurpassed chasteness as well as accuracy; they have become *cyclopedic.* And, in the absence of either dryness or superfluity, no other treatise can in our view, be held superior to this. Its value is heightened by the addition of a number of new and good illustrations, to those now common to all such works. Of the 296 wood engravings scattered through Dr. Draper's volume, 68 are named as "by the author;" a goodly number in these days of compilation. A number of them have been obtained by the aid of *microscopic photography;* the process having, as it is stated, "been so far improved by the author as to be rendered very available for these uses." Nearly all of the copied engravings, also, have been obtained through the intervention of photographs.

The work is divided into two Books; the first of which, under the head of Statical Physiology, comprises twenty-five chapters, on the "Conditions of Life"—giving the history of all those functions usually called organic (except reproduction), and also those of the organs of sensation, of the brain, and of animal motion. The second book contains, as "Dynamical Physiology," eight chapters, on "the Course of Life." Development, Reproduction, Sleep and Death, and the Influence of Physical Agents upon organized beings, fill all of these but the concluding chapter, which is upon "Social Mechanics;" viewing history as a corollary to Physiology. In regard to the method of this whole arrangement, we shall have a few words to say presently. Let us, first glance through the volume, to see how it fulfils its second indication or purpose as a Compend of the essential facts of the Science of Physiology.

In this regard, we shall find it well brought up to the time, and containing a considerable amount of matter which is comparatively new and original, both in opinion, and in observation and experiment. By the omission, for the most part, of long argumentative discussions, and of historical accounts and references upon mooted points, the author has obtained space, in a volume of moderate size, for more, of real importance, than is often compressed into the same bulk without the loss of completeness.

The opening subject of the work is that of the conditions of life—"air, water, and combustible matter." In discussing the "receipts and waste" of the body, besides the facts ordinarily stated, tabular views are given; as Physiological Standard Tables, of the diurnal ingesta, secretions and excretions of a man of the weight of 140 lbs.; and also of the same items compared to the weight of the body as 1000 parts.

The history of Digestion, given in chapters III and IV, is that which is warranted by the latest observations—including those of Lehmann and others, upon the usual presence of lactic acid in the gastric juice, recently confirmed (as reported in the *Examiner*, July, 1856) by Dr. F. G. Smith, in his experiments upon Alexis St. Martin.

In the chapter upon Absorption, it is stated that "the action of the villus is doubtless more complicated than is generally represented; for the organic fibre-cells it contains give to it the power of executing *rythmic motions.*" Peyer's glands are regarded by Dr. Draper as belonging to the absorbent rather than the digestive apparatus; many lacteal vessels proceeding from them, "as may be very plainly observed during digestion." The elaboration of fibrin from albumen, and the development of the chyle-corpuscles (embryos of the blood-cells) are considered to be the two principal purposes of the absorbent system, including its glands. The view of J. Simon and some other British physiologists and pathologists, that fibrin is an effete and not a plastic substance, is objected to by Professor Draper.

The subject of capillary attraction has long been a favorite one with our author; especially as to the mode in which it affects the movement of the blood. The following propositions are laid down:

"1st. If the force of attraction of the particles of a solid for those of a liquid, be not equal to *half* the cohesive force of the latter for each other, the liquid will refuse to pass through a pore of that solid substance, and, in a capillary tube consisting of it, will be depressed below its hydrostatic level.

2nd. If the force of attraction of the particles of a solid for those of a liquid exceeds *half* the cohesive force of the latter for each other, but is not equal to the whole force, the liquid will pass through a pore of that solid substance, and, in a capillary tube of it, will rise above its hydrostatic level.

3d. If the force of attraction of the particles of a solid for those of a liquid exceeds the whole cohesive force of the latter, chemical union between them ensues."

Endosmosis and exosmosis are correctly explained as identical with capillary attraction, except in the additional power given by the presence of a fluid *at the ends* of the fine tubes which the membrane or porous septum represents. The law commonly stated, that the stronger current in endosmotic transmission is always from the lighter to the denser liquid, is denied. "Its error is readily shewn by many simple illustrations. The relation of specific gravity has nothing whatever to do with the action." (p. 107.)

The contribution rendered by Professor Draper to the theory of the circulation of the blood, through his experiments and observations upon capillary phenomena, is familiar to every physiological student. It may be interesting, nevertheless, to recall, in his own language, the principal basis of the opinion, associated with his name, that capillary power is *the* agency by which the blood is circulated through the whole frame, as the sap is moved in plants. It is (so far as we can quote without the diagram) as follows:

"If two liquids communicate with one another in a capillary tube, for the substance of which they have affinities of different intensities, movement will ensue—the liquid having the highest affinity will occupy the tube, and may even drive the other before it. The same effect will ensue in a porous structure. Thus, let *b*, *b*, be a capillary tube of any kind, which is occupied conjointly by two liquids, *a* and *v*, meeting each other in its middle; *a* having a high and *v* but little affinity for the substance of which the tube consists, *a* will occupy the tube, pressing out *v* before it. Of course, it is to be understood that the liquids *a* and *v*, respectively, communicate with reservoirs that can furnish them a necessary supply. The various phenomena described under the designation of endosmosis are experimental illustrations of the same kind. Now it is precisely relations of this kind that are observed in the case of the circulating and nutritive juices of all organic beings." "The juice which is about to nourish a part has for that part a certain affinity, but, with the accomplishment of that nutrition, the affinity is at once lost. Thus, for instance, in the systemic circulation, the parts to be nourished have a certain affinity for the arterial blood; they take from it whatever their purposes require, and, that done, the relation at once ceases; the blood, become venous, has lost its hold upon them, and is pressed off. We may conveniently describe this effect as a pressure of the unchanged upon the changed liquid."

This principle, variously applied, is supposed by Dr. Draper to account for the *whole circulation* of the blood, with some trifling aid, only, from the heart, equalized in turn by the arteries.

It is impossible to acknowledge so much as this, notwithstanding the importance of the principle, (with some slight modification) as explaining *one* of the moving agencies of the circulation.

The modification which reflection suggests as necessary, is, we think, easily made intelligible.

Let us recall the three laws of capillary action, quoted upon a previous page, and, in view of them, compare the experiment of the tube and two liquids, with the circumstances of blood-circulation and nutrition.

There is this difference; that, while, in the tube experiment, no combination is supposed to take place between the liquid and the walls of the tube, in the case of the blood flowing through a capillary vessel, we must notice that—1st, the governing attraction is *not* for the walls of the vessel, but for something *outside* of them; and 2nd, that a *combination takes place* between the particles attracting and those attracted. The latter particles are therefore being constantly *withdrawn* in a current or currents through the sides of the vessel.

The case of the experiment with the tube and fluids comes under the second of the laws laid down by Prof. Draper on p. 105 of his work; the attraction of the particles of the tube for those of *one* of the fluids exceeding *half* the cohesive force of the latter, but not equalling the whole of it; and thus causing it to rise above its hydrostatic level, and even carrying the other fluid before it.

But, in the case of the blood-vessel whose plasma is passing out, laterally, for nutrition or secretion, a *double* capillarity is observed; that of the *capillary tube* itself, and that of its *permeable walls*. The chemical combination, also, which is a part of the nutrition, or secretion, &c., occurring, makes it a case under the 3d law, above alluded to, when, the force of attraction of the particles engaged exceeding their whole cohesive force, chemical combination, instead of hydrostatic impetus, ensues. The traction is lateral, not impulsive. The reticular arrangement of the capillaries varies incessantly its direction, it is true; but still, in every case, the power exerted is only, in regard to the current in the vessels, sufficient to act, (as lateral suction might do, to a less degree, by removal of pressure), in facilitating the approach of particles of the blood *to* the capillary region in which extravasation is taking place. No important addition can thus be afforded to the impetus of the venous circulation returning toward the heart. We are anxious to make this, if possible clear; since there is a certain amount of boldness in thus proposing to modify a doctrine, now so generally accepted. A rude illustration may elucidate our meaning further.

Suppose that, in the middle of a passage-way between two parallel ropes, two files of men meet each other, going in opposite directions. Suppose, also, that of those going eastward all use traction by the ropes; while those who go westward are all *hand-bound*. There will then be a case somewhat like that of the experiment of Dr. Draper, with the tube and two liquids; one of which, having attraction for the walls of the tube, pushes the other before it.

But if, on the other hand, we suppose that, instead of traction, *extraction* be used by other men *outside of the ropes*, lifting out of the passage one after another of those who go in one direction, we have then the case of nutritive affinity clearly represented: the effect upon the opposing current or file, being one of *arrest*, only, not of backward propulsion, to any extent. The relative importance of the power derived from such a source as one of the mechanical agencies of the circulatory system, would seem to be diminished, not at all destroyed, by these considerations.

We might say more. Dr. Draper is of opinion that "the arterialization of the blood in the lungs is the cause of the circulation in man. I consider the circulation as the consequence of respiration." Its subordinate actions are "all referable to one primordial act, and that is, the exposure of the blood to the air."

And yet, it is understood, that the oxygen we breathe is not permanently, but loosely, if at all, combined in the blood, but is carried by the red discs or blood-cells, and freely given off to the tissues. In the words of our author, "the generation of carbonic acid is commonly referred to the soft tissues. Doubtless much originates in this way." "That nothing analogous to combustion," (that is, active chemical combination,) "occurs in the lungs, is proved by their temperature, which is not higher than that of other parts of the system."

And yet, we are called upon to believe that the affinity of the blood in the capillaries of the lungs for oxygen (although not sufficient to produce any active combination,) is, at last, *the cause*, not merely one of the contributing causes of the circulation! Again, the portal circulation is accounted for, with all its exceptional character, by a chemico-dynamic relation between the liver and portal blood. Yet Dr. Draper himself holds that "the bile simply transudes from the blood, and that the cells of the lobules have *no special relation to it* beyond this, that it oozes past their interstices, or, perhaps, *by physical imbibition*, finds access to their interior." Certainly there must be here some inadvertency, if not inconsistency.

The author states, that the action of the heart is "limited to *bringing the blood to the arterial origin of the capillaries*, and beyond that point its action may be considered not to extend." (pp. 145–7.) Yet, the absolute force which the left ventricle exerts for the propulsion of the blood into the systemic arteries, is mentioned (upon p. 139) as being equal to 13 pounds; that of the right ventricle about half as much. *What becomes* of all this force? It certainly cannot be all spent in dilating the arteries, which, by their muscularity, would re-bound without loss, but rather with gain of power. The blood, moreover, is not *to be brought*, with each impulse of the heart, to the capillaries, but, being already there, the *column only* has to be impelled. This has been overlooked.

In fine, the many phenomena adduced (upon p. 144) to show the necessity of a capillary power, apply equally well, in most cases, to that of an *arterial* impulsion; and are to be accounted for by the joint influence of *both* these systems acting as supplementary to the heart. We must be understood as not denying the existence of a modifying power exerted upon the circulation in the capillaries, dependent upon the relations of the blood to the tissues; but only as deprecating the exaggerated estimate of its *relative* importance, into which Dr. Draper, with many other recent writers, seems to have fallen.

The chapter upon Respiration presents, in addition to the ordinary facts, some curious statements in regard to interstitial movements exhibited by *solids*, as well as by liquids and gases; besides some very interesting investigations by the author upon the occurrence of gaseous diffusion, without diminution, under a pressure of fifty atmospheres; and experiments upon the amount of watery vapor of respiration, by his son, Dr. J. C. Draper, of New York. It was proved, by these last experiments, that the quantity of water exhaled varies directly with the *number of respirations*; not being diminished in proportion, as is the case with carbonic acid, when they are increased in rapidity.

The influence of diurnal variations of temperature upon the condition of the body, in health and disease, is noticed in the succeeding chapter upon Animal Heat.

Much stress is also laid upon a probable mode of action of the nervous system, which has not received all the attention it deserves. It is that of its controlling, not only animal heat, but many other changes in the living body, by affecting the *allotropic state* of its component elements, whether simple or compound. Berzelius and Faraday have made great account of allotropism in inorganic bodies; it remains to be definitively applied to the organic.

"There are some well-known facts in natural philosophy, which throw a flood of light on this obscurity. If a piece of pure zinc be placed in a glass of acidulated water beside a piece of copper, so long as the metals are kept apart, no action whatever ensues; but if a conducting thread is laid from one to the other, the zinc instantly begins to oxidize, clouds of hydrogen gas bubbles rise from the copper, and the thread becomes at once red-hot and magnetic. On lifting the communicating thread, all these actions cease; on restoring it they instantly recur. We think we explain them by saying they are all due to the decomposition of water by the zinc. But why was the zinc passive when alone, and why did it assume this activity when merely touched by another metal? Does not all this serve to show that substances may be, as it were, in a quiescent state, and, on the application of what may, perhaps, seem the most insignificant cause, may suddenly assume activity, and forthwith satisfy their chemical affinities?

"I have endeavored to prove that allotropism is the true cause of many of the obscure facts which we meet with in the animal mechanism." "We may conclude that, so far as tissue destruction is concerned, the nervous system possesses a governing or controlling power; that, by keeping parts in states answering to the passive and active conditions of inorganic chemistry, it can suspend the action of the respired oxygen or permit it to take effect." "It is also possible that these states of activity or passivity are likewise impressed upon the oxygen itself, since that gas is no exception to the rule; it also exhibits allotropism. Its passive state is Priestley's oxygen, its active is ozone."

These suggestions are important, and flow naturally from many recent investigations. Would not the term *dynamization* be, perhaps, more convenient than "active allotropism," or allotropic states? Ozone is dynamized oxygen. The nervous system gives a dynamic energy to particles otherwise inert. We may conceive that in the blood, this intensity of the affinities of all its elements may be wrought up to its highest pitch; and that in this consists part of the effect of the assimilating organs. Portal blood is liquid food; arterial blood, fluid tissue.

The view of Dr. Draper in regard to the action of the liver in the secretion of bile, has been already alluded to. Upon the broad subject of secretion, his statement is this: "Perhaps we should not be very far from the truth if we considered all those secretions in which the materials are in a state of retrograde metamorphosis, or in a descending career, as arising by mere filtration, and those which are ascending to a higher grade as due to cell agency; between the two there being an intermediate class, the phase of which is stationary, and in which cells may or may not be necessarily involved; as, for instance, the transmutation of one fat into another, or the preparation of sugar from the albumenoid bodies." It is added, that "the more profoundly we study the composition and constitution of the secreted fluids, and the more accurately we understand the function of secretion itself, the less are we disposed to invoke the agency of cell-life, and to rely the more on the ordinary mechanical act of strainage."

Bile, it is said, is increased as to its amount of fluid by calomel, but its solid constituents diminished. Carbonate of soda diminishes both. These statements are not ordinarily made, and may not be universally acknowledged. The fact that the resinous matter of bile is found in the stools after mercurials, although not commonly present, has been stated by several observers.

The fact of the manufacture of *sugar* by the hepatic cells, is fully admitted. And yet, sugar, being vegetative, can hardly be called a product of progressive metamorphosis. Why then, might not similar cells *produce bile*, perhaps as a complementary result? It is, indeed, supposed, by Dr. Draper, that the coloring matter of the bile is derived from hæmatin, the blood-cells being disintegrated in the liver and in the spleen.

As to the functions of the latter organ, the views of Prof. Kölliker are adopted, and no allusion is made to the admirable essay of Mr. Gray, in which, results are stated which were obtained by experiments and observations on a large scale, and with the utmost care, proving that the function of the spleen is to regulate both the *quantity* and *quality* of the blood.

We cannot but think it straining a theory not too firmly established, to class the emission of milk from the mammary glands among the *excretions*, in immediate succession to the consideration of the urine.* Not to dwell long upon this point, if even casein (as kyestein) and the other ingredients of the mammary secretion are pre-existent in the blood (which is denied), how could *corpuscular forms*, milk corpuscles, exist in it, without at least some agency from *cell-life?* They certainly cannot be *filtered* out of the gland.

The existence of nucleated cells upon the Malpighian tuft or

* Chap. XII.

coil has been proved by Dr. Isaacs; a drawing from whose paper, read before the New York Academy of Medicine, is given, confirming Bowman's views of the structure of the kidney.

Passing by a chapter on "Nutrition and Decay," as more connected with the second Book, we come next to the Functions of the Nervous System.

Some exceedingly interesting remarks are made upon the supposed action of the vesicular nervous centres as "registering ganglia," and as reservoirs of force.

"There cannot be a doubt that the registry of impressions involves an actual structural change in the ganglion, which is of a permanent character. These changes may be rudely and imperfectly illustrated by experiments, such as I published years ago, of which the following may be taken as examples: If, on a cold, polished piece of metal, any object, as a wafer, is laid, and the metal then be breathed upon, and, when the moisture has had time to disappear, the wafer be thrown off, though now upon the polished surface the most critical inspection can discover no trace of any form, if we breathe upon it a spectral figure of the wafer comes into view, and this may be done again and again. Nay, even more; if the polished metal be carefully put aside where nothing can deteriorate its surface, and be so kept for many months, (I have witnessed it even after a year) on breathing again upon it, the shadowy form emerges; or, if a sheet of paper on which a key or other object is laid be carried for a few moments into the sunshine, and then instantaneously viewed in the dark, the key being simultaneously removed, a fading spectre of the key on the paper will be seen; and if the paper be put away where nothing can disturb it, and so kept for many months, at the end thereof, if it be carried into a dark place and laid on a piece of hot metal, the spectre of the key will come forth. In the case of bodies more phosphorescent than paper, the spectres of many different objects which may have been in succession laid originally thereupon, will, on warming, emerge in their proper order.

I introduce these illustrations for the purpose of showing how trivial are the impressions which may be thus registered and preserved. Indeed, I believe that a shadow never falls upon a wall without leaving thereupon its permanent trace—a trace which might be made visible by resorting to proper processes. All kinds of photographic drawing are in their degree examples of the kind."

The chapter on the Brain appears, to us, one of the least satisfactory in the volume; considering the amount of knowledge added to our stock since the days of Hartley. It would seem that a certain dread of "metaphysical speculations" has deterred the author from attention to inquiries which might rather with correctness be called "physchological inductions."

An implied assent is given to the doctrine of Gall and Spurzheim, "that particular regions of the brain are devoted to special functions, and that by an inspection of the exterior of the cranium mental peculiarities may be detected." No opinion is given (as might have been expected) upon the value of the inductive evidence asserted by the phrenologists, as to the detailed localization of these special cerebral organs. We have only the rather loose (for a volume of "Positive Science") allusion, as follows, to one series of them: (p. 546.) "It *has been asserted*, that the moral and intellectual peculiarities which woman presents when compared with man, are distinctly traceable to the phrenological preponderance of the moral over the intellectual portions of the brain."

The opinion of Descartes (advocated also by Brougham, Carpenter, and others,) that insects are, even in the most complicated of their instinctive actions, automatic, is disputed. We have no hesitation in accepting the views of the highly distinguished authorities above named upon this subject. We are told by Professor Draper (p. 609), that "they are not automata, since they are under the influence of the cephalic ganglia as the registers of past impressions." A just view of comparative psychology proves that this does not alter the case, in regard to automatism. Nor does the exercise of intelligence. Instincts are either executive or impulsive; in the lowest animals the former; in the higher, the latter, almost alone.

We may refer to the admirable chapters on the Nervous System in Dr. Carpenter's Treatises (Human and Comparative Physiology) for the best discussion of this subject extant. There it is proved, especially upon the basis of Dr. Laycock's investigations, that our ideas of automatic or reflex action must not be limited even by instinct. *Cerebro-reflex* action occurs, even in man, to an extent, and with an importance, which only needs suggestion to be confirmed by every one's experience.

Looking upwards, through the animal series, from the amæba toward man, we shall find (as true positive philosophy throws the *onus probandi* upon the side of *volition* as an improbable postulate) that a careful exclusion will leave no evidence of actions in the invertebrate, no, nor in the vertebrated animal, which cannot be ascribed to automatic influences, acting through a complex muscular, ganglionic, or cerebral mechanism, until we come to *man* himself. And, in man, the evidence of a different power, of choice, of will, comes from *consciousness*, not from observation. Looking upon other men, we would have no proof of it. Man *is* an automaton, not only as regards his spinal system, but so long as he is governed, as many men are, by unrestrained instincts, propensities, ideas: while, in the highest efforts of genius, especially in its æsthetic developments, there are evidences of an automatic nature, although under the control of the will, as clear as in the simplest case of reflex action. We dwell upon these things, not because they are not familiar, but to regret that Dr. Draper has preferred to ignore them; and to express the opinion, that they are of great importance, in relation to that philosophy of accountability and morality, which he has repeatedly spoken of in terms of the highest respect.

Passing on, we find in the work before us a very clear and interesting account of the physiology of the organs of sense. The general view taken of the "triple function" of the internal acoustic apparatus, is, that 1st, the *drum* is for the measurement of intensity of sound; 2d, the *cochlea* for the recognition of wave-length (pitch?); 3d, the *semi-circular canals* for the appreciation of the quality of sounds. The last of these conclusions is the least satisfactorily established. The argument would seem to be at least equally strong in favor of either of the other hypotheses; the older one, that the semicircular canals have relation to the direction of sounds,—or that of Roget and Professor Jackson, that they serve to exhaust or arrest *waste* undulations, and to prevent their repercussion upon the ear.

As to the eye, several very interesting suggestions are discussed. Thus,

"There has been a difference of opinion as respects the actual screen upon which the images form. Some of the early optical writers regarded the black pigment as being that receiving surface, an opinion which has been universally abandoned, the function having been of late attributed to the retina, but, as it appears to me, on totally insufficient grounds. The arguments against the retina, both optical and anatomical, are perfectly unanswerable. During life it is a transparent medium, as incapable of receiving an image as a sheet of clear glass, or the atmospheric air itself; and its sensory surface is, structurally, its exterior one; that is, the one nearest the choroid coat. But the black pigment, from its perfect opacity, not only completely absorbs the rays of light, turning them, if such a phrase may be used, into heat, no matter how faint they may be, but also discharges the well-known duty of darkening the interior of the eye, and therefore preventing indistinctness from the straying of the rays of light. Perfection of vision requires that the images should form on a mathematical superficies, and not in the midst of a transparent medium. The black pigment satisfies that condition, the retina does not."

We have also a theory of the *rationale* of vision, as a function of the retina. On the strength of his own experiments, and those of Melloni, the author states that, although the prism indicates otherwise, the yellow or brightest ray is the hottest, and that the warming powers of the others follow in the order of their luminous intensity. And, in his opinion, the act of vision commences in this local disturbance of temperature. The club-shaped particles of Jacob's membrane are tactile organs, which communicate to the sensory surface of the retina the condition of temperature of the black pigment. But this communication of a variety of temperature implies a variation in the waste and repair of the retina itself; the physical change being thus converted into a physiological equivalent in the metamorphosis and destruction of a nervous tissue. Now the essential condition of the chemical activity of a ray of light, whether for photographic or calorific effects, is absorption. A surface like the black pigment is capable of absorbing all the rays alike. The impression arising from the disturbed condition of the retinal vesicles is carried by the optic tubules to the chiasm of the optic nerves; and, we might add, to the tubercula quadrigemina, as the central ganglia of sight. Objections may be raised to parts of this theory; but the problem is too abstruse for the present article. Professor Draper recognizes the undulatory theory of light upon another page, in saying that "it amounts only to a different mode of stating the same effect, since, as I have shown (Lond. and Edin. Philosoph. Magazine, May, 1851), all chemical changes accomplished in material substances are occasioned by the establishment of vibratory motions therein, and Ampère has already demonstrated that all the phenomena of heat may be explained upon the doctrine of the vibrations of the constituent molecules of bodies."

A somewhat analogous theory to the above is presented in regard to the physiological action of the muscular fibre; to the effect that "muscular contraction is the *physical result* of *muscular disintegration.*" Rhythmic, as well as other contractions are thus explained, upon a principle which we must now pass by. One or two yet more important points remain for discussion, the omission of which would be inappropriate.

We have observed, that the whole domain of Physiology has, by our author, been mapped out into Statical and Dynamical. We are informed that, since physiology is to be viewed as a branch of natural philosophy, much advantage is to be expected, both present and future, from introducing, in this manner, the methods familiar to writers on mechanics.

But, we cannot think, a physicist would see, without surprise, upon a page connected with *Statical* Physiology, full descriptions of the mechanism of *locomotion*, standing, walking, and running, while, upon the opposite one, a *Dynamical* series of chapters begins, of which one is wholly occupied with *Sleep* and *Death.* The terms, it would seem to us, though we say it with deference, lack the accuracy which should recommend an innovation of this kind, otherwise than as a mere novelty. Care is especially required in transferring the method of one science to another, since *similarity* of treatment, only, not identity, is possible, where the subjects are not the same. "If the case was clear," writes the astute Comte, "in regard to the intrusion of the geometers into physics, it is yet more so with regard to the intrusion of the physicists into Biology; on account of the *more essential difference in the nature of the two sciences.*"

Is not Statical Physiology simply *Anatomy*, the study of the body as it *exists*, not as it *acts?* It is true, that a *movement*, a career, occurs in growth, and may be called dynamic; but this is true *also*, and more "in the method familiar to physics," in the movements of the body in mass, and in the reactions which belong to the properly animal functions of the organism. An ambiguity is thus created, rather to a disadvantage than otherwise.

De Blainville's subdivision of Biology into zootomy, or anatomy,—zootaxy, or classification, and dynamical biology or physiology, is a better one: the latter being then divisible into the functions of Organic and those of Animal Life. The collateral advantages sought for by Professor Draper could be thus as fully obtained, at least, without any doubtful terminology. We are the more surprised at the classification he has chosen, from the manner in which it stands related to the views he holds of Life and of Development.

These are the purely physico-chemical views. Not Boerhaave, Lamarck, Lehmann, or Moleschott, could go farther. Although provisional use is made of the "elementary conception" of a "plastic power," and its "apparent evidence, from a superficial consideration,"—this is corrected afterwards, by an explicit denial of the existence of anything more than the ordinary physical agencies of inorganic matter.

We ought to justify such assertions by free quotation; and this is easily done. On page 500, we have it as a general statement, that

"Structural differentiation is to be received as the cause of functional differentiation. The former, in every instance, arises from the changed circumstances under which cells are being generated."

Again, (p. 253):

"It appears to me that many instances of nutrition, growth, and development can only be explained by admitting, as a great and fundamental principle in physiology, that, the primordial germ being in all instances alike, its mode of development will depend on the physical agents and conditions to which it is exposed."

The following might well please Lamarck, or the author of the "Vestiges of Creation." (p. 506).

"Starting from a solitary cell, development takes place; and, according as extraneous forces may be brought into action, variable in their nature and differing in their intensity, the resulting organism will differ. If such language may be used, the aim of nature is to reach a certain ideal model or archetype. As the passage toward the ideal model is more or less perfectly accomplished, form after form, in varied succession arises. The original substratum or material is, in every instance alike; for it matters not what may be the class of animals or of plants, the primordial germ, as far ar investigation has gone, is in every instance the same. The microscope shews no difference, but, on the contrary, demonstrates the identity of the first cell, which, if it passes but a little way upon its forward course, ends in presenting the obscure cryptogamic plant, or, if it runs forward toward reaching the archetype, ends in the production of man. The diversity of form that is eventually presented, depends then, not upon the constitution or aspect of the primitive cell, but upon the influence of the many surrounding agencies to which it is exposed." "Not only is the primordial cell in all instances the same, but the first stages of its career are, in all instances, identical; and this, whether we consider it in the lowest or the highest cases, belonging either to the vegetable or the animal kingdom." "This career of development applies equally to the case of any individual animal, or any race of animals. Thus, man himself, in succession, passes through a great variety of forms, from the condition of a simple cell; these forms merging by degrees into one another, the form of the serpent, of the fish, of the bird, and this not only as regards the entire system, but also as regards each one of its constituent mechanisms—the nervous system, the circulatory, the digestive."

We have, therefore, each one of us, had a *narrow escape*, depending "altogether on extraneous circumstances," from turning out "an armadillo, a flying-fish, or a stag-beetle!" We need hardly stop now to say, that those who have contributed the most to the science of embryology as it is, make a different statement from the above: to the effect that, in every case, the law of development from the *general to the special* is the only one that can be affirmed; no real correspondence ever existing, for example, between the embryo of Man and the completed type or form of any other class, in any of the systems which have been referred to; the real resemblance being only in the *embryoes* of the different classes and types, in the gradual process of differentiation.*

Looking farther into Dr. Draper's views, we are met, unexpectedly, by a somewhat different train of thought. He remarks (p. 549), that

"That active agent which was first laid in a fold of the germinal membrane was not annihilated when its type of life was changed to placental and therefore aquatic respiration. It withstood the shock when again, after a due season, it was suddenly made to breathe the air. Arrived at the mature condition, the nature of every one of its organs has changed. Through all these losses and changes, the immaterial principle has passed unchanged, and even gathering strength. In the broadest manner that a fact can be set forth, we see herein the complete subordination of matter, and the *enduring character of spirit.*"

It will be hard not to confound this with ultra vitalism: or rather, with the doctrine of the "unconscious soul," now held by Dr. Laycock, and approved even by the philosophical Morell. But, let us look back to our former quotations. Coming to the point, in all earnestness, we ask *how about cabbages?* If the agent, or spirit, "is *laid in the primordial cell*," identical exactly for all species, animal or vegetable, and depending upon "external

* Vide Carpenter's Princip. Com. Physiol., Am. Ed., p. 125.

conditions" only, for its development into the one type or another, *what becomes of the spirit*, when these conditions favor a lower instead of a higher type? That we are not mistaken in Dr. D.'s meaning, as regards the identity of the "immaterial principle" alluded to with the soul of man, is shown upon p. 25, where it is said to be an important duty with the physiologist,—regretting the use of such terms as "mind, intellect, vital principle, spirit," which confuse the popular ideas,—to prevent, as far as he can, such expressions from "frittering away the great truth that, as there is but one God in the universe, so there is but one spirit in man."

In the discussion of the subject of Reproduction, the same error (in our belief) has polarized the judgment of our author to some degree. Ambiguity appears to be created by the mode of use of the word "individuality." *Unity* would have been a better term; which is compatible with *dividuality;* as illustrated in multiplication by *gemmation*, which is *always exhaustible.* This is acknowledged by Dr. D., in accepting Carpenter's modification of Steenstrup's views. The organism *begins as a unit*, and differentiates;—into few cells or a million,—like or unlike,—dissevered or together,—but still always (in the language of the work before us) "expanding from one common or single centre;" not built up from foreign materials imposed from without: unity first, multiplication afterwards; and this in all cases with a limit, until renewed by a generative reproduction.

The idea conveyed in the terms "plastic power" and "vital force" is, as we have seen, especially objected to by Dr. Draper. We have not space now to go into an elaborate disquisition upon them. It may be said, however, that those who now authoritatively use the latter, and some modification of the former, (as Liebig and Carpenter), do so not in a "metaphysical sense," but in one entirely compatible with the analogies of physics, and the methods of natural philosophy. The expressions of the work of Dr. Draper himself would justify them. Why else does he devote a whole Book to *dynamical* physiology, whose topic is simply the *career* of an organic germ—if that career has no governing and especial influence of its own? Discarding, as modern science does, the fiction of "entities"—and granting with Dr. Draper that the course of life, the motion of development, is not an "agent," but an "operation,"—it is yet a peculiar operation. If there is reason for designating (however, provisionally) by the name of *heat*, the physical source of certain expansive and other movements of matter,—by that of *light*, that of certain others,—and of gravitation, of others,—we must allow that there is the *same* reason for differentiating that power which affects the mode in which other physical agents react upon the animal or vegetable germ, to so important a degree as life is known to do. We may accept the phrase "momentum of development;" a momentum, however, never exhausted until death. We must object to the term "self-origination" as applied to the organic cell (p. 494) in any case, as quite unphilosophical. But the physical illustrations adduced by Dr. Draper for a somewhat different purpose, may be made to serve ours equally well. "The career of an organism recalls the flight of a heavy projectile, thrown upwards;" and we give the name of life-force to its impulse, derived, no doubt, from an "antecedent impression." Plants and animals are, in truth, "nothing more than temporary states" of material, transitory results (like the flame of a lamp) of some "original impression," that of parentage, acting with the external agencies of nature. In the words of M. Comte, however,* we must never forget the "dualism" between the aggregate of external actions, and the action of the organism itself: the organism and the medium being both indispensable factors in a true dynamic physiology.

Error exists, it appears to us, in the assumption, too commonly made, that, because there is no *perceptible* difference between germ and germ, of different species, we can therefore predicate that no difference exists. We have no graduated *vitometer* to measure life-temperature, to small degrees, or to estimate germinal capacity. We must consider ourselves very much in the dark, therefore, upon the whole subject. A dogmatic negation would be as great an evil as a "middle aged" hypothesis. The "positive" authors, of late times, do not always remember this. They decry old theories; but they invent new ones.

Dr. Draper has ranged himself under the flag of the "new"

* Positive Philosophy, translated by H. Martinean; p. 357.

Positive Philosophy. We are entitled, therefore, to regret, that we are unable to see any advantage the world has gained from its advent in the 19th century,—not ascribable, in reality, to the "antecedent impression" of Lord Bacon in the 16th. If it differs from this in anything, it is to a disadvantage: introducing a sort of rule of the *guillotine;* attempting to "purify the republic" of science by the removal of all suspected ideas; whereas the true remedy for all evils, social or scientific, is to be found in the due gradation of authorities, not in their destruction. If the hill of Sisyphus be the acknowledged type of all human efforts after absolute knowledge, we may admire the philosophic mind which accepts with calmness the necessity; but not the defiant spirit which, while it denounces all that is past, utters an anticipatory "eureka" with every breath.

We have no such animadversion to make on the work before us; but we may confess that some sentences in the Preface gave rise to these reflections, by a not unnatural suggestion. Thus:

"We live in a period of difficulty. Metaphysical Philosophy has lost its hold upon the human mind. The uncertainties, contradictions and emptiness of the English, Scotch, French, and German schools are manifest. Already the belief is wide spread that their barrenness of result and consequent worthlessness are the necessary incident of their method of investigation, and that we must look to some *wholly new* system as a guide to the truth on the topics they have had under consideration. That guide is Positive Science!"

To aid in removing Physiology,—"which, alone, of all the great departments of knowledge, still retains the metaphysical conceptions of the Middle Ages,"—from Speculative and attaching it to Natural Philosophy, is stated to be one of the great objects of Dr. Draper's work. But, may we not respectfully ask, are not Valentin and Lehmann, Longet and Bernard, Owen and Carpenter, Agassiz and Burnett, and scores of others, already cultivators of *positive physiological science?* If so, the removal would seem to be already accomplished; certainly is not now to be begun.

Let us, in conclusion, glance, with great pleasure, at those chapters in the work which we have not yet alluded to, but which can be only named. They are chapters II, VII, and VIII, of Book II; containing a most *admirably* written series of observations upon the influence of physical agents upon vegetable and animal life, including that of Man ; in which, amongst many other points of great interest, the Unity of the Human Species is well maintained. We have, in fine, a concluding chapter, which would do credit to the most erudite writer upon "Social Mechanics ;" comprising a Physiological view of History, from the earliest authentic dates to modern times.

Upon the whole, we rise from the survey of this work, of which, if we have "nothing extenuated," we have at least "nought set down in malice," with an impression of great power and value. No ordinary book would repay half the care and study required for even these imperfect remarks.

While, therefore, we would see, with pleasure, in a future edition, some alterations, both of arrangement and opinion, we are satisfied that "Draper's Physiology" will take an important place at once : and will add one more to the number of American text-books, which may be placed side by side with the best of those obtained from abroad. If for no other consideration, we would thank Professor Draper for his work ; and thanks are also due to the publishers, for the good print, good paper, and excellent style altogether, with which it does credit to their press.

---

*Human Physiology.* By Robley Dunglison, M. D., LL. D., Professor of the Institutes of Medicine in Jefferson Medical College, Philadelphia ; Vice-President of the American Philosophical Society, etc., etc. *With five hundred and thirty-two Illustrations. Eighth edition, revised, and enlarged. In two volumes.* Philadelphia : Blanchard & Lea. 1856.

It affords us much pleasure to be able to announce the appearance of the eighth edition of the above work. The very short period in which the previous issues have been exhausted, exhibits, in a very flattering light, the high estimation in which the work is held by the profession. In preparing the present edition, "no pains have been spared to make the work a complete expression of the science of the day." This statement our own examination of the work enables us to confirm ; every page of it almost testifying to the author's industry in culling from various quarters and sources all that was valuable in the physiological contributions to science of the last few years. The careful and scrutinizing spirit exhibited by the writer, when investigating mooted questions; the extensive information he possesses of general science in almost every department, and the clear and happy style in which he presents his views, render his physiology one of the most reliable and attractive works in our language.

To the practitioner and general reader, we can heartily recommend it as an excellent resumé of the present state of physiological science. As a text-book for the student, we think it has no superior in our language, and for this object we presume it was chiefly if not expressly written.

---

*New Remedies: With Formulæ for their Preparation and Administration.* By ROBLEY DUNGLINSON, M. D., Professor of the Institutes of Medicine, etc., in the Jefferson Medical College of Philadelphia. *Seventh edition with numerous additions.* Philadelphia: Blanchard & Lea. 1856.

Among the new remedies (either so-called from their being lately discovered, or from their being employed in a manner unknown before) described in the present edition are apiol, caffein, carbazotic acid, cauterization and catheterism of the larynx and trachea, cedron, cerium, chloride of bromine, chloride of iron, chloride of sodium, cinchonicine, cod-liver olein, congelation, eau de Pagliari, galvanic cautery, hydriodic ether, hyposulphite of soda and silver, inunction, iodide of sodium, nickel, permanganate of potassa, phosphate of lime, pumpkin, quinidia, rennet, saccharine carbonate of iron and manganese, santonin, tellurium and traumaticine.

The authority from which his information was obtained is given by the author, in every instance, at the bottom of the page, by which means the reader, anxious to avail himself of fuller information on any subject, is enabled to refer at once to the original article. The sources of information are exceedingly various, the materials being taken from English, German, French and American authorities. Proverbial as is the author's industry, the labor he must have undergone in collecting so great a mass of facts, cannot but excite the astonishment of every one who knows how tedious and toilsome such research is. The style in which the work is written is flowing and perspicuous, and the opinions given are marked by the author's usual judgment and caution. Independently of the information it contains, the book is an agreeable and readable one; and to all who are desirous of becoming acquainted with the properties and applications of the many new remedial agents of the last few years, we take great pleasure in recommending it.

---

*Obstetrical Tables.* By Dr. Pajot, Agrégé Professor to the Faculty of Medicine, Paris. *Translated from the French, and arranged by* O. A. Crenshaw, M. D., and J. B. McCaw, M. D., Richmond, Va. *With three additional Tables on the Mechanism of Natural, Unnatural and Complex Labors.* By Nathan P. Rice, M. D., New York. Richmond: Enquirer Book and Job Press. 1856.

Dr. Pajot's is a name well known to obstetrical science, and this work, though but a summary, is, by the admirable clearness and methodical arrangement of the tables, (five in number,) a most valuable addition to our other means of instruction on some of the most difficult and dangerous obstetrical complications.

Table first is devoted to the signs of pregnancy, with the functional derangements thence arising, together with a tabulated review of the touch, palpation, auscultation and percussion as means of diagnosis.

The second table is a classification of the deformities of the female pelvis in connection with child-birth; the causes of malformation, with the indications and treatment.

The third table is a synopsis of the treatment of hæmorrhage.

The fourth is an admirable summary of the subject of turning, with the difficulties and complications attending the operation.

The fifth and last of this series is devoted to the application of the forceps, divided into three stages. First, the introduction and placing of the branches. 2d, the articulation of the blades, and 3d, the stage of extraction.

The translators have considerably enhanced the value of the work by adding three excellently arranged tables, compiled by our countryman, Dr. Nathan P. Rice, of New York, on the subjects of Natural, Unnatural and Complex labors.

Drs. Crenshaw and McCaw deserve the thanks of the profession for the great care and correctness with which they have translated and arranged these tables. Dr. McCaw's summary of the causes and treatment of post-portem hæmorrhage concludes the work, and renders the series complete in every particular. The work is beautifully got up, and, as to paper, type, &c., unexceptionable. We recommend it with confidence to students and practitioners as a useful addition to their libraries.

---

*A Review of the Present State of Uterine Pathology.* By JAMES HENRY BENNETT, M. D., &c. Philadelphia: Blanchard & Lea. 1856.

The object of this little work is a critical consideration of the several objections urged by Drs. Lee and West against the existence and pathological importance of inflamatory ulceration of the neck of the uterus. The leucorrhœa theory, the syphilis theory, the ovarian theory and the displacement theory are all of them severally reviewed, also, and found wanting. As an exposition of the author's peculiar views upon uterine pathology, the corretness of which time and experience have fully satisfied him, and, as an able argumentative discussion of the doctrines held by other writers, we heartily recommend it to our readers.

---

*The Physician's Visiting List, Diary aud Book of Engagements for* 1857. Philadelphia: Lindsay & Blakiston.

The above contains an almanac, a table of signs, a table of poisons, with their antidotes, a table for calculating the period of utero-gestation, blank leaves for visiting list, memoranda, addresses of patients, memoranda of wants, and of obstetric and vaccination engagement. Copies are prepared for twenty-five or fifty patients, and, when needed, will be printed for one hundred patients. We have used the visiting list for several years past, and can confidently recommend it to the profession as an excellent diary and book of engagements.

